# 분만실에 근무하는 조산사의 직무수행과 소명의식이 직무만족도에 미치는 영향

**DOI:** 10.4069/kjwhn.2020.02.27

**Published:** 2020-03-12

**Authors:** Geum Ah Jung, Moon Jeong Kim

**Affiliations:** Department of Nursing, Pukyong National University, Busan, Korea; 부경대학교 간호학과

**Keywords:** Nurse midwives, Work performance, Religious personnel, Job satisfaction, 조산사, 직무수행, 소명의식, 직무만족

## Abstract

**Purpose:**

The purpose of this study was to investigate work performance and calling as determinants of job satisfaction among nurse midwives.

**Methods:**

The participants of this study were registered nurse midwives who had worked in the delivery room for more than 6 months. Data were collected by face-to-face interviews, postal mail, and mobile devices. Subjects completed self-report questionnaires from July to August 2017. The dataset was analyzed using descriptive statistics, the independent t-test, analysis of variance, the Pearson correlation coefficient, and multiple regression.

**Results:**

The mean score for job satisfaction was 3.42±0.45. Among the sub-factors, income had the lowest score (2.67±0.72) and management of delivery had the highest score (3.81±0.66). Job satisfaction was significantly different according to marital status (t=2.25, *p*=.028), residential area (t=2.43, *p*=.016), and cause of job satisfaction (F=4.54, *p*=.012). Job satisfaction showed a significant positive correlation with work performance (r=.29, *p*<.001) and calling (r=.57, *p*<.001). The correlation between work performance and calling was also positive and statistically significant (r=.32, *p*<.001). Purpose and meaning (β=.48, *p*<.001) and marital status (β=–.15, *p*=.025) significantly influenced job satisfaction. The model developed in this study explained 45% of variation in job satisfaction.

**Conclusion:**

Nurse midwives’ job satisfaction may be enhanced by entrusting them with professional roles and tasks. Above all, it is necessary to develop and provide programs that help nurse midwives connect their jobs with the meaning and purpose of their lives.

## Introduction

### 연구 필요성

출산 과정에서 조산사의 전문적인 지지는 진통제 사용과 회음절개술 및 회음 손상, 기계분만, 제왕절개술의 비율을 낮추고 출산 경험에 대한 만족도를 높이며[1] 산부의 분만 자신감을 높이는[2] 등, 조산사의 역할에 대한 긍정적인 보고들이 많다. 그러나 2016년 임상 조산사의 OECD (Organization for Economic Cooperation and Development) 회원국 평균은 인구 천명당 0.35명이었으며 이중 한국은 0.02명으로 자료가 가용한 22개 국가 중 최하위로 나타났다[3].

출산에 대한 조산사들의 긍정적인 기여에도 불구하고 이들의 근무 현황이나 근무만족도에 대한 경험적 증거는 매우 부족하다. 간호사가 자신의 직무에 만족할수록 직무 몰입이 높아지고 동기가 유발되어 조직의 성과가 높아지므로[4] 분만실에서 일하는 조산사의 근무만족도는 산부와 가족의 만족도와 병원의 조직 성과에 중요한 영향을 미치리라 생각되지만, 이에 대한 근거가 매우 부족하므로 조산사의 직무만족 요인을 설명하는 다양한 요인에 대한 탐색이 이루어질 필요가 있다.

전문 간호사의 경우 해당 자격과 동일한 분야에서 전문직 간호를 수행할 때 직무만족도가 높으며[5], 개업 조산사의 경우에도 전문적인 역할 수행이 높을수록 직무만족도가 증가하는 것으로 알려졌다[6]. 조산사의 전문적인 역할 수행은 개업 조산사의 경우 의료법의 제한으로 역할 수행 빈도가 낮았으며[6], 병원에 고용된 조산사는 개업 조산사보다 더 낮은 것으로 보고되었다[7]. 2015년 병원에서 근무하는 조산사는 641명, 조산원에서 근무하는 조산사는 39명으로 대부분의 조산사가 병원에서 근무하고 있다[8]. 즉, 병원에 고용된 상태이나 분만실에 근무하고 있어 전문직 역할 수행이 높을 것으로 예상되는 조산사를 대상으로 전문적인 직무수행이 근무만족도에 미치는 영향을 파악해 볼 필요가 있다.

한편 일을 소명으로 여기는 사람들은 다른 사람들에 비해 삶의 만족과 직무만족이 높은 것으로 보고되었다[9]. 이러한 소명의식은 일에 있어서 자신의 역할을 깨닫고, 그 속에서 의미와 목표를 추구하며, 그로 인해 사회나 온 인류를 위한 선에 긍정적인 영향을 미치려는 태도를 의미한다[10]. 간호사에게 있어서도 소명의식은 직무만족에 영향을 미쳤으며[11] 의료기관에 종사하는 간호사와 의사의 소명의식은 직무 스트레스와 이직 의도 사이를 조절하는 효과가 있었다[12]. 선행연구의 결과를 미루어 볼 때 소명의식은 조산사의 직무만족과도 관련이 있을 것으로 생각되므로 이에 대한 파악이 필요하다. 이에 본 연구는 분만실에 근무하는 조산사를 대상으로 직무수행 정도와 소명의식이 직무만족도에 미치는 영향을 파악하고자 한다.

### 연구 목적

본 연구는 조산사의 직무수행 정도와 소명의식이 직무만족도에 미치는 영향을 파악하기 위한 것이며 구체적인 목적은 다음과 같다.

1) 대상자의 일반적 특성과 근무 관련 특성을 파악하고, 대상자의 특성에 따른 직무만족도의 차이를 파악한다.

2) 대상자의 직무수행과 소명의식, 직무만족의 정도를 파악한다.

3) 대상자의 직무수행과 소명의식, 직무만족의 상관관계를 파악한다.

4) 대상자의 직무수행과 소명의식이 직무만족도에 미치는 영향을 파악한다.

## Methods

Ethics statement: This study was approved by the Institutional Review Board of Pukyong National University (1041386-20170621-HR-016-3). Informed consent was obtained from the subjects.

### 연구 설계

본 연구는 분만실에 근무하는 조산사의 직무수행 정도와 소명의식이 직무만족도에 미치는 영향을 조사한 횡단적 조사연구이다.

### 연구 대상

본 연구는 대한조산협회에 등록된 조산사로서 연락처가 확인되는 자 가운데 분만실에 근무하고 있으며 조산사 수습기간을 제외한 임상 경력이 6개월 이상인 자를 대상으로 하였다. G*Power 3.1.2 프로그램을 이용하여 유의수준(α)=.05, 검정력(1–β)=0.85, 중간 효과 크기(f)=0.15, 예측변수 12개로 설정하였을 때, 다변량 회귀분석을 위한 최소 표본의 크기는 140명이었다. 85%의 회수율을 가정하여 161부의 설문지를 배부하였고 153부가 회수되었으나 응답이 누락된 설문지를 제외한 후 149부의 자료를 최종 분석에 활용하였다.

### 연구 도구

#### 대상자의 특성

대상자의 특성을 파악하기 위하여 일반적 특성으로는 연령, 결혼상태, 학력, 종교, 월 소득, 거주지 등 6개 항목을 포함하였다. 근무 관련 특성에는 임상 경력, 분만실 근무 경력, 분만 개조 여부, 직업만족 이유 등 4개 항목을 포함하였다. 직무수행과 소명의식, 직무만족도의 측정을 위한 도구들은 이메일로 개발자의 승인을 받은 후 사용하였다.

#### 조산사 직무수행 정도

분만실에 근무하는 조산사의 직무수행 정도를 측정하기 위하여 조산사 국가시험 문항 개발 기준안 연구[13]에서 제시한 직무수행 항목을 이용하였다. 본 도구는 산전관리 9문항, 분만관리 11문항, 산후관리 8문항, 신생아관리 3문항, 자기계발 3문항 등 5개 하부영역과 34개 문항을 포함하고 있다. 그 가운데 본 연구의 대상자인 병원 분만실에 근무하는 조산사에게는 해당하지 않는다고 판단된 임신 진단하기, 회음부 절개하기, 산모 심폐소생술하기, 여성건강 관리 등의 4개 문항은 조사에서 제외하였다. 4점 Likert 척도로 ‘경험 없다’ 0점, ‘자주 한다’에 3점을 부여하였으며 총점의 범위는 0–90점이었다. 점수가 클수록 해당 직무를 자주 수행함을 의미하며 도구의 신뢰도는 개발 당시 Cronbach’s alpha 값이 .92 이었고, 본 연구에서는 .93이었다.

#### 소명의식

소명의식은 Shim과 Yoo [14]의 한국판 소명의식 척도를 사용하여 측정하였다. 본 도구는 초월적 부름, 목적‧의미, 친사회적 지향 등의 3개 하부요인과 12개 문항으로 구성되어 있다. 4점 Likert 척도로 ‘전혀 아니다’ 1점, ‘매우 그렇다’에 4점을 부여하였으며, 총점의 범위는 12–48점으로 점수가 클수록 소명의식이 높음을 의미한다. 도구의 신뢰도는 개발 당시 Cronbach’s alpha값은 .85이었고, 본 연구에서는 .86이었다.

#### 직무만족도

조산사의 직무만족도는 Kim [6]이 사용한 도구로 측정하였다. 본 도구는 전문지식, 조산업무, 보수, 근무환경, 업무감독 등 5개 하부영역, 20개 문항으로 구성되어 있다. 5점 Likert 척도로 ‘전혀 아니다’ 1점, ‘매우 그렇다’에 5점을 부여하였으며 총점의 범위는 20–100점이고 점수가 클수록 직무만족도가 높음을 의미한다. 개발 당시 도구의 신뢰도 Cronbach’s alpha 값은 .99이었고, 본 연구에서는 .88이었다.

### 자료 수집 방법

자료수집은 2017년 6월부터 8월까지 이루어졌다. 자료 수집을 시작하기 전에 연구자가 소속된 부경대학교의 연구윤리심의위원회의 승인을 받은 후 방문과 우편, 온라인으로 자료를 수집하였다. 방문 조사를 위하여 대한조산협회에서 실시하는 조산사 보수교육 일정을 파악한 후, 사전에 보수교육기관 관계자에게 연구의 목적과 방법에 대해 설명하고 허락과 협조를 구하였다. 보수교육 당일 대상자들에게 구조화된 설문지를 배부하고 보수교육 종료 후 설문지를 수거하였다. 그리고 조산사 양성기관에 전화로 협조를 구한 뒤 우편으로 설문지와 소정의 선물을 동봉하여 보낸 후 완성된 설문지를 우편으로 회수하였다. 온라인 조사를 위하여 대한조산협회의 허락과 협조를 얻은 후 등록 조산사의 전화번호를 얻었다. 대상자에게 문자 메시지로 연구 안내문을 전송하고 연구 참여에 동의하면 링크를 통해 온라인 조사 사이트로 연결할 수 있게 하였다. 설문지 작성에 소요된 시간은 평균 15–20분이었다. 모든 참가자들에게 소정의 답례품을 제공하였다.

### 자료 분석

본 연구에서 수집된 자료는 IBM SPSS Statistics for Windows, ver. 21.0 (IBM Co., Armonk, NY, USA)을 이용하여 통계 분석을 실시하였다.

1) 대상자의 일반적 특성과 근무 관련 특성은 빈도와 백분율로, 대상자의 특성에 따른 직무만족도의 차이는 independent t-test와 ANOVA로, 사후비교는 Scheffé’s test로 분석하였다.

2) 대상자의 직무수행과 소명의식, 직무만족의 정도는 평균과 표준편차로 구하였다.

3) 대상자의 직무수행 및 소명의식의 하부요인과 직무만족도의 상관관계는 Pearson’s correlation coefficient로 분석하였다.

4) 대상자의 직무만족도 영향요인은 다중회귀분석으로 분석하였다.

## Results

### 대상자의 특성과 그에 따른 직무만족도의 차이

본 연구 대상자의 일반적 특성을 보면, 연령은 평균 42.2±8.3세로 40대가 47.0%로 가장 많았고 39세 이하가 30.2%, 50대 이상이 22.8%였다. 대부분의 대상자가 결혼한 상태였고(77.2%), 4년제 대학교와 대학원을 졸업한 대상자는 69.8%였다. 종교는 기독교가 가장 많았고(45.6%) 없는 경우는 28.9%였다. 월 소득은 300만 원 미만이 51.7%로 가장 많았고 대부분 비수도권에 거주하였다(77.9%). 대상자의 일반적 특성에 따른 직무만족도의 차이를 보면, 결혼상태(t=2.25, *p*=.028)와 거주지(t=2.43, *p*=.016)에 따른 차이가 유의하였고, 연령(F=0.89, *p*=.412), 교육수준(F=0.05, *p*=.952), 종교(F=0.18, *p*=.910), 월 수입(F=0.21, *p*=.890)에 따른 차이는 유의하지 않았다. 즉 배우자가 없는 대상자와 수도권에 거주하는 대상자의 직무만족도가 높았다

대상자의 근무 특성을 살펴보면, 임상 경력은 11–20년이 41.6%로 가장 많았고, 분만실 근무 경력은 10년 이하가 58.4%로 가장 많았으며 대부분 분만 개조를 하지 않았다(77.9%). 현 직업에 만족하는 이유로는 ‘보람과 긍지’가 52.3%로 가장 많았고, ‘자아실현’은 37.6%였다. 근무 특성에 따른 직무만족도의 차이를 살펴보면, 현 직업에 만족하는 이유에 따른 차이가 유의하였고(F=4.54, *p*=.012), 임상 경력(F=3.02, *p*=.052), 분만실 근무 경력(F=0.87, *p*=.422)이나 분만 개조 여부(t=0.58, *p*=.565)에 따른 차이는 유의하지 않았다. 즉 현 직업에 만족하는 이유가 ‘급여 등 기타’인 대상자보다 ‘보람과 긍지’인 대상자의 직무만족도가 더 높았다(Table 1).

### 직무수행과 소명의식, 직무만족의 정도

직무수행의 평균은 3점 만점에 1.97±0.50점이었고, 하부요인별로는 산전관리가 2.26±0.57점으로 가장 높았으며, 산후관리 2.17±0.67점, 분만관리 2.01±0.43점, 자기계발 1.70±0.80점, 신생아관리 1.70±0.92점이었다. 소명의식의 평균은 4점 만점에 2.97±0.38점이었고 하부요인별로는 목적‧의미가 3.12±0.45점으로 가장 높았고 친사회적 성향이 2.96±0.43점, 초월적 부름이 2.84±0.51점이었다. 직무만족도는 5점 만점에 평균 3.42±0.45점이었으며 하부요인별로 살펴보면 분만업무가 3.81±0.66점으로 가장 높았고, 전문지식 3.51±0.64점, 근무환경 3.44±0.56점, 업무감독 3.24±0.68점, 보수가 2.67±0.72점 순으로 나타났다(Table 2).

### 직무수행과 소명의식, 직무만족도의 상관관계

직무수행(r=.29, *p*<.001)과 소명의식(r=.57, *p*<.001)은 직무만족도와 유의한 양의 상관관계를 나타내었다. 그리고 직무수행과 소명의식 간에도 유의한 양의 상관관계(r=.32, *p*<.001)를 보였다. 직무수행과 소명의식의 하부요인들과 직무만족도 간의 상관관계는 Table 3과 같았다.

### 직무만족도 영향요인

직무수행과 소명의식이 조산사의 직무만족도에 미치는 영향을 파악하기 위하여 다중회귀분석을 실시하였다. Durbin–Watson 지수가 1.95로 오차항들 간에 서로 독립적임을 확인하였고, 독립변수들의 공차한계는 0.46–0.94로 0.1보다 컸으며, 분산팽창지수는 1.07–2.19로 10 미만이었으므로 다중공선성의 문제가 없는 것으로 판단하였다.

대상자의 특성 가운데 직무만족도에 유의한 차이를 나타낸 명목변수인 결혼상태(1=결혼), 거주지(1=수도권)와 현 직업에 만족하는 이유(1=보람과 긍지)를 더미변수 처리한 후 회귀식에 투입하였다. 그리고 직무만족도와 유의한 상관관계를 나타낸 직무수행과 소명의식의 하부요인들도 함께 투입한 결과, 병원 분만실 조산사의 직무만족도를 설명하는 회귀모형은 유의하였으며(F=13.09, *p*<.001) 설명력은 45%였다.

독립변수들이 직무만족도에 미친 영향은 목적‧의미(β=.48, *p*<.001), 결혼상태(β=–.15, *p*=.025) 순으로 강하였다. 즉 직업에 대한 목적‧의미를 높게 인식하고 미혼인 조산사의 직무만족도가 높은 것으로 나타났다(Table 4).

## Discussion

본 연구는 조산사의 직무수행과 소명의식, 직무만족도의 정도를 파악하고, 직무수행과 소명의식이 직무만족도에 미치는 영향을 파악하기 위해 진행하였다. 주요 연구결과를 중심으로 논의하고자 한다.

본 연구에서 조산사의 직무수행 정도는 3점 만점에 평균 1.97점이었고 하부요인별로는 산전관리가 2.26점으로 가장 높았으며, 산후관리 2.17점, 분만관리 2.01점, 자기계발과 신생아관리가 각각 1.70점이었다. 동일한 도구로 측정한 결과는 아니지만 선행연구에서 조산원 조산사의 직무수행 정도는 5점 만점에 평균 4.05점(2.43/3점)이었고, 하부요인별로는 산전관리 4.03점(2.42/3점), 산후관리 4.65점(2.79/3점), 분만관리 2.78점(1.67/3점), 신생아관리 4.50점(2.70/3점), 자기계발 4.18점(2.51/3점)으로[7] 분만관리를 제외한 모든 영역에서 본 연구보다 수행빈도가 높았다. 병원 분만실에 근무하는 조산사들이 조산원 조산사들에 비해 직무수행 정도가 낮게 나타난 것은 병원에서는 분만 직후 신생아를 신생아실로 이동시키고, 분만 후 한두 시간 안에 산모를 산과병동으로 이동시키므로 신생아관리와 산후관리를 제공할 기회가 적기 때문인 것으로 보인다. 조산원은 입원부터 퇴원까지 한 공간에서 연속적인 돌봄을 제공하므로 분만 후에도 산후관리와 신생아관리 등 직무수행의 빈도가 높은 것으로 생각된다[15]. 다른 한편, 조산사의 직무수행 측정도구가 다양한 영역의 직무를 수행할수록 높은 점수를 받도록 고안되었기 때문에, 직무가 분만관리에 집중된 분만실 조산사의 경우 상대적으로 점수가 낮게 평가되었을 것으로 생각된다. 따라서 후속연구에서는 조산사의 전문적인 직무수행 정도를 측정할 때 수행빈도와 수행시간을 함께 측정할 것을 제안한다.

본 연구에서 소명의식은 4점 만점에 평균 2.97점으로 하부요인별로는 목적‧의미가 평균 3.12점(74/100점)으로 가장 높았고 친사회적 지향 2.96점, 초월적 부름 2.84점이었다. 같은 도구를 사용한 중소병원 간호사의 소명의식은 5점 만점에 평균 3.13점(63/100)점이었고 목적‧의미 3.33점, 친사회적 지향이 3.25점, 초월적 부름이 2.82점이었다[11]. 본 연구와 선행연구[11]에서 소명의식은 조산사와 간호사의 직무만족도 영향요인으로 보고되고 있으므로, 조산사와 일반 간호사의 소명의식에 체계적인 차이가 있는지 비교연구가 필요하다. 더 나아가 개인이나 조직의 어떤 특성이 소명의식에 영향을 미치는지 파악한다면 간호사 하위집단의 특성을 이해하고 그에 맞는 소명의식 증진을 위한 중재 개발에 도움이 되리라 생각한다.

본 연구에서 직무만족도는 5점 만점에 평균 3.42점이었다. 하부영역별로는 분만업무에 대한 만족이 3.81점(76.2/100점)으로 가장 높았고 보수에 대한 만족이 2.67점(53.4/100점)으로 가장 낮아, 본 연구의 대상자들이 전문적인 업무나 위치에 만족하는 한편 보수에 대해서는 상대적으로 만족하지 못하고 있음을 알 수 있다. 분만관리는 조산사에게 있어 가장 전문성이 발휘되는 직무이기 때문에 이에 대한 직무만족도가 높은 것으로 해석된다. 이러한 결과는, 조산원에 근무하는 조산사들이 이전에 병원 분만실에서 근무할 때는 조산사의 정체성에 대한 회의를 많이 느꼈지만 조산원에서 분만 중심의 업무를 수행하면서 조산사로서의 자부심과 책임감을 느끼고 즐겁게 업무에 임하게 되었다는 질적연구의 결과[16]와 맥을 같이 한다. 병원에서 간호인력을 배치할 때 조산사를 분만업무와 관련된 부서, 즉 분만실에 배치하는 것을 적극적으로 고려할 필요가 있다.

본 연구에서 신생아관리를 제외한 조산사의 직무수행은 직무만족도와 양의 상관관계를 나타내었다. 본 연구의 결과는 전문 간호사가 해당 자격 분야와 동일한 분야에서 전문 간호 직무를 수행하는 경우가 그렇지 않은 경우에 비해 직무만족도가 높았다[5]는 선행연구의 결과를 지지하고 있다. 이는 조산사의 직무만족도를 높이기 위해서는 조산사를 전문 간호 직무를 수행할 기회가 많은 부서에 적극적으로 배치해야 할 필요성을 시사한다. 저출산으로 출산 여성은 감소하였으나 고령 임신으로 인한 합병증의 위험이 높아졌기 때문에 고위험 임부부터 고위험 신생아에 이르기까지 집중적인 관리가 필요해졌다. 고위험 산모 신생아 통합치료센터(Maternal Fetal Intensive Care Unit, MFICU)에 근무하는 간호사는 체계적인 교육을 받지 않은 상태에서 MFICU에 발령받는 경우가 많아 전문적인 역량을 갖추지 못하고 직무수행의 어려움을 느껴 이직을 고려하는 사례가 많은 것으로 보고되고 있다[17]. 국가가 발급하는 조산사 면허증을 갖추고 전문 지식과 기술을 갖춘 조산사를 MFICU의 인력으로 적극 활용한다면 MFICU에서의 간호의 질이 높아지는 동시에 조산사의 직무만족도를 높여 전문 인력을 안정적으로 활용할 수 있을 것으로 기대된다.

본 연구에서 직무수행 가운데 자기계발이 직무만족도와 가장 높은 상관관계를 보였다. 본 연구에서 4년제 대학교 졸업 이상의 학력을 가진 조산사는 69.8%었으며 이는 대학병원 일반 간호사의 57.5%와 비교했을 때 높은 수준으로[18], 분만실 조산사들의 자기계발 욕구가 비교적 높음을 알 수 있다. 그러나 분만실 간호사는 근무경험에서 자기계발의 어려움을 가장 크게 호소하였는데[19] 이는 소속 기관의 자기계발 여건이 충분하지 않은 상황과 관련된 것으로 보인다. 2018년 현재 조산사의 18%는 종합병원에, 54%는 병·의원에, 28%는 조산원에 근무하고 있는 실정으로[8], 대부분의 조산사가 규모가 작은 기관에 소속되어 있어 자기계발의 기회가 제한적일 수 있다. 임상실습의 현장지도자는 실습지도가 성장을 위한 자극제로 작용하여 임상수행 능력을 향상시키기 위한 자발적 노력으로 이어지고 자기계발을 위한 교육의 기회를 찾게 된다고 하였다[20]. 많은 병원들이 간호학과와 실습 협약을 맺고, 조산사들이 현장지도자 경험을 통해 자기계발의 동기를 얻으며, 병원이 조산사의 자기계발 기회를 지원한다면 함께 성장하는 동력이 될 수 있을 것이다.

본 연구에서 조산사의 소명의식의 모든 하부요인은 직무만족도와 양의 상관관계를 나타내었다. 그리고 직무만족도 영향요인을 파악하기 위하여 다중회귀분석을 실시한 결과 소명의식의 하부요인 가운데 목적∙의미와 결혼상태(기혼)의 영향이 유의하게 나타났다. 간호사를 대상으로 한 선행연구에서 목적‧의미는 재직의도의 영향요인으로 나타났는데[21], 본 연구의 결과는 이와 맥락을 같이 한다. 직무만족도에 있어서 초월적 부름이나 친사회적 지향보다는 목적‧의미의 영향이 강한 이유는 오늘날 개인주의적 가치관과 관련 있어 보인다. 일이 신이나 사회의 부름에 의한 것 또는 타인이나 공동체의 이익을 위한 것이라는 태도는 개인의 밖을 지향한 것이지만, 일의 의미와 일을 통해 개인적 목적을 실현해 나가는 것을 중시하는 것은 개인의 내부를 향한 태도이기 때문이다. 소명을 가진 사람들이 자신의 일에 확신을 가지고 몰입하며 만족할 수 있는 것은 그들이 인지하고 있는 일에 대한 의미와 목적이 내적 동기로 작용하기 때문이다[22]. 다행히 소명의식은 안정적이고 변화가 어려운 기질적인 속성이 아니라 발전과 관리가 가능한 상태적인 속성으로 알려져 있다[14]. 조산사의 소명의식, 특히 일을 삶의 목적이나 의미와 연결시키려는 목적‧의미의 태도를 강화하는 훈련을 주기적으로 제공한다면 조산사의 직무만족도를 높이는 데 도움이 될 것이다.

본 연구에서 배우자가 있는 대상자일수록 근무만족도가 유의하게 낮았다. 기혼 간호사는 엄격한 조직체계 아래에서 개인에게 주어진 막중한 책임 때문에 일-가정 간의 균형을 유지하면서 다중 역할을 수행하는 데 많은 어려움을 느낄 수 있다. 일-가정 갈등이 있는 기혼의 여성 간호사에서 가족 친화 제도 및 상사의 지지가 일과 삶의 균형에 긍정적인 방향으로 영향을 미친 결과는 상사의 지원이 직장 내 사회적 지지의 주요한 원천이 될 수 있음을 말해준다[23]. 그리고 일-가정 갈등은 가정 내 역할을 더 많이 한다고 느낄수록, 본인의 근로시간이 길수록, 동거하는 미취학 자녀의 수가 많을수록, 본인의 가사 및 돌봄 시간이 길수록 커졌다[24]. 탄력근로제나 직장 내 보육시설 운영 등의 가족 친화적인 제도를 실시한다면 기혼 조산사의 근무만족도를 향상시킬 수 있으리라 생각한다.

지역 간 의료의 심각한 불균형을 고려할 때 전국적인 표본이 아니라 영남 지역에 치중된 표본을 사용한 점은 본 연구의 제한점이므로 연구의 결과를 전체 병원 분만실에 근무하는 조산사에게 일반화하는 데 신중을 기할 필요가 있다. 병원 조산사와 조산원 조산사의 직무에 많은 차이가 있음에도 불구하고 조산원 조산사 위주의 직무수행 도구로 직무수행을 측정한 점도 한계점이 될 수 있을 것이다.

## Conclusion

본 연구를 통해 병원 분만실에 근무하는 조산사를 대상으로 직무수행 정도와 소명의식이 직무만족도에 미치는 영향을 파악하고자 하였다. 소명의식 가운데 목적∙의미가 병원 분만실에 근무하는 조산사의 직무만족도 영향요인으로 나타난 바, 조산사들에게 직업을 통해 삶의 가치와 목적을 실현하고자 하는 태도를 증진시킬 기회를 제공한다면 직무만족도 개선에 효과가 있을 것으로 생각된다. 본 연구는 병원 분만실에 근무하는 조산사의 소명의식과 결혼상태가 직무만족도 영향요인임을 밝힘으로써 조산사의 직무만족도를 높이기 위한 전략을 마련하는 데 유용한 시사점을 제공한 점에 의의가 있다.

본 연구의 결과를 바탕으로 다음을 제언한다. 첫째, MFICU에 근무하는 조산사와 일반 간호사의 직무수행과 직무만족도를 비교하는 연구가 필요하다. 둘째, 기혼 조산사의 일-가정 양립이 직무만족도에 미치는 영향을 파악해 볼 필요가 있다. 셋째, 조산사의 소명의식, 특히 목적‧의미를 고양하는 프로그램을 개발하고 직무만족도에 대한 효과를 검증하는 연구를 제안한다. 넷째, 병원 분만실 조산사의 직무를 분석할 필요가 있다.

## Figures and Tables

**Table 1.** Differences in job satisfaction by general and work characteristics (N=149)

Characteristic	Categories	n (%)	Mean±SD	t/F (*p*)
*General*				
Age (year)	≤39	45 (30.2)	3.38±0.35	0.89 (.412)
	40–49	70 (47.0)	3.41±0.41	
	≥50	34 (22.8)	3.49±0.37	
				
Marital status	Yes	115 (77.2)	3.38±0.47	2.25 (.028)
	No	34 (22.8)	3.55±0.35	
Education	College	45 (30.2)	3.40±0.45	0.05 (.952)
	University	66 (44.3)	3.42±0.45	
	≥Master	38 (25.5)	3.43±0.48	
Religion	Protestantism	68 (45.6)	3.42±0.49	0.18 (.910)
	Buddhism	22 (14.8)	3.36±0.41	
	Catholicism	16 (10.7)	3.45±0.35	
	None	43 (28.9)	3.44±0.46	
Monthly income	≤299	77 (51.7)	3.39±0.48	0.21 (.890)
(×10,000, KRW)	300–399	47 (31.5)	3.44±0.45	
	400–499	17 (11.4)	3.47±0.34	
	≥500	8 (5.4)	3.38±0.49	
Residential area	Capital	33 (22.1)	3.58±0.44	2.43 (.016)
	Non-capital	116 (77.9)	3.37±0.44	
*Work-related *				
Length of nurse experience (year)	≤10	36 (24.2)	3.48±0.39	3.02 (.052)
	11–20	62 (41.6)	3.31±0.48	
	≥21	51 (34.2)	3.50±0.44	
Length of midwife experience (year)	≤10	87 (58.4)	3.39±0.44	0.87 (.422)
	11–20	39 (26.2)	3.42±0.51	
	≥21	23 (15.4)	3.53±0.40	
Deliver babies	Yes	33 (22.1)	3.46±0.50	0.58 (.565)
	No	116 (77.9)	3.41±0.44	
Cause of job satisfaction	Sense of worth and pride^a^	78 (52.3)	3.50±0.48	4.54 (.012)
	Self-actualization^b^	56 (37.6)	3.38±0.38	
	Salary, etc.^c^	15 (10.1)	3.14±0.42	a>c^†^

KRW: Korean won.

† Scheffé test.

**Table 2.** Scores of research variables (N=149)

Purpose and meaning	Range	Mean±SD
Work performance	0.34–2.89	1.97±0.50
Antepartum care	0.10–3.00	2.26±0.57
Intrapartum care	0.23–3.00	2.01±0.43
Postpartum care	0.00–3.00	2.17±0.67
Neonatal care	0.00–3.00	1.70±0.92
Self-improvement	0.00–3.00	1.70±0.80
Calling	2.08–4.00	2.97±0.38
Transcendence	1.25–4.00	2.84±0.51
Meaning and purpose	2.00–4.00	3.12±0.45
Prosocial intention	2.00–4.00	2.96±0.43
Job satisfaction	2.20–4.65	3.42±0.45
Management of delivery	2.25–5.00	3.81±0.66
Professional knowledge	1.67–5.00	3.51±0.64
Work environment	1.75–4.80	3.44±0.56
Clinical supervision	1.33–5.00	3.24±0.68
Income	1.00–4.50	2.67±0.72

**Table 3.** Correlations among work performance, calling, and job satisfaction (N=149)

Variable	r (*p*)									
	WP	APC	IPC	PPC	NM	SI	Call	Tran	PM	PSI
APC	.62 (<.001)	1								
IPC	.66 (<.001)	.49 (<.001)	1							
PPC	.83 (<.001)	.44 (<.001)	.52 (<.001)	1						
NM	.77 (<.001)	.17 (.034)	.40 (<.001)	.63 (<.001)	1					
SI	.71 (<.001)	.38 (<.001)	.27 (.001)	.20 (<.001)	.36 (<.001)	1				
Call	.32 (<.001)	.17 (.037)	.22 (.006)	.19 (.019)	.22 (.007)	.36 (<.001)	1			
Tran	.18 (.027)	.04 (.626)	.22 (.008)	.09 (.294)	.13 (.108)	.21 (.012)	.81 (<.001)	1		
PM	.34 (<.001)	.23 (.005)	.19 (.018)	.23 (.005)	.12 (.015)	.39 (<.001)	.86 (<.001)	.50 (<.001)	1	
PSI	.29 (<.001)	.17 (.035)	.14 (.085)	.18 (.033)	.23 (.005)	.30 (<.001)	.84 (<.001)	.46 (<.001)	.67 (<.001)	1
JS	.29 (<.001)	.24 (.003)	.18 (.033)	.21 (.012)	.12 (.162)	.35 (<.001)	.57 (<.001)	.36 (<.001)	.62 (<.001)	.48 (<.001)

APC: antepartum care; Call: calling; IPC: intrapartum care; JS: job satisfaction; NM: neonatal management; PM: purpose and meaning; PPC: postpartum care; PSI: prosocial intention; SI self-improvement; Tran: transcendence; WP: work performance.

**Table 4.** Factors influencing job satisfaction (N=149)

Purpose and meaning	B	SE	β	t	*p*
(Constant)	1.25	0.28		4.52	<.001
Marital status^†^	–0.15	0.07	–.15	–2.26	0.025
Residential area^†^	0.1	0.07	.09	1.34	0.183
Cause of job satisfaction^†^	0.3	0.24	.08	1.22	0.226
Antepartum care	0.04	0.06	.05	0.59	0.558
Intrapartum care	0.02	0.08	.02	0.21	0.833
Postpartum care	–0.02	0.05	–.03	–0.34	0.737
Self-improvement	0.06	0.04	.10	1.35	0.178
Transcendent	0.07	0.07	.08	1.02	0.308
Purpose and meaning	0.48	0.09	.48	5.32	<.001
Prosocial intention	0.11	0.09	.11	1.27	0.208
R²=.49; adjusted R²=.45; F(*p*)=13.09 (<.001)					

† Dummy variable references were marital status (married), residential area (capital), and cause of job satisfaction (sense of worth and pride).
